# A derived dryolestid mammal indicates possible insular endemism in the Late Jurassic of Germany

**DOI:** 10.1007/s00114-021-01719-z

**Published:** 2021-05-16

**Authors:** Thomas Martin, Alexander O. Averianov, Julia A. Schultz, Achim H. Schwermann, Oliver Wings

**Affiliations:** 1 Section Palaeontology, Institute of Geosciences, Rheinische Friedrich-Wilhelms-Universität Bonn, Bonn, Germany; 2 Department of Theriology, Zoological Institute of the Russian Academy of Sciences, Saint Petersburg, Russia; 3 LWL-Museum für Naturkunde, Westfälisches Landesmuseum mit Planetarium, Münster, Germany; 4 Natural Sciences Collections, Martin-Luther-Universität Halle-Wittenberg, Halle (Saale), Germany

**Keywords:** Dryolestidae, *Hercynodon*, Insular endemism, Jurassic, Langenberg Quarry, Mesozoic mammal

## Abstract

The Langenberg Quarry near Bad Harzburg has yielded the first Jurassic stem therian mammal of Germany, recovered from Kimmeridgian (Late Jurassic) near shore deposits of a palaeo-island within the Lower Saxony Basin of the European archipelago. The new stem therian is represented by one lower and three upper molars. *Hercynodon germanicus* gen. et sp. nov. is attributed to the Dryolestidae, a group of pretribosphenic crown mammals that was common in western Laurasia from the Middle Jurassic to the Early Cretaceous. The new taxon is characterised by small size, a reduced cusp pattern in the upper molars lacking a metacone, and enhancement of the shearing crests paracrista and metacrista. Phylogenetic analysis identified *Hercynodon* gen. nov. as sister taxon of *Crusafontia* from the Lower Cretaceous (Barremian) of Spain. Both taxa belong to an endemic European clade of dryolestids, including also *Achyrodon* and *Phascolestes* from the earliest Cretaceous (Berriasian) of England. Despite its greater geological age, *Hercynodon* gen. nov. is the most derived representative of that clade, indicated by the complete reduction of the metacone. The discrepancy between derived morphology and geological age may be explained by an increased rate of character evolution in insular isolation. Other insular phenomena have earlier been observed in vertebrates from the Langenberg Quarry, such as dwarfism in the small sauropod *Europasaurus*, and possible gigantism in the morganucodontan mammaliaform *Storchodon* and the pinheirodontid multituberculate mammal *Teutonodon* which grew unusually large.

**Supplementary Information:**

The online version contains supplementary material available at 10.1007/s00114-021-01719-z.

## Introduction

**Fig. 1:**
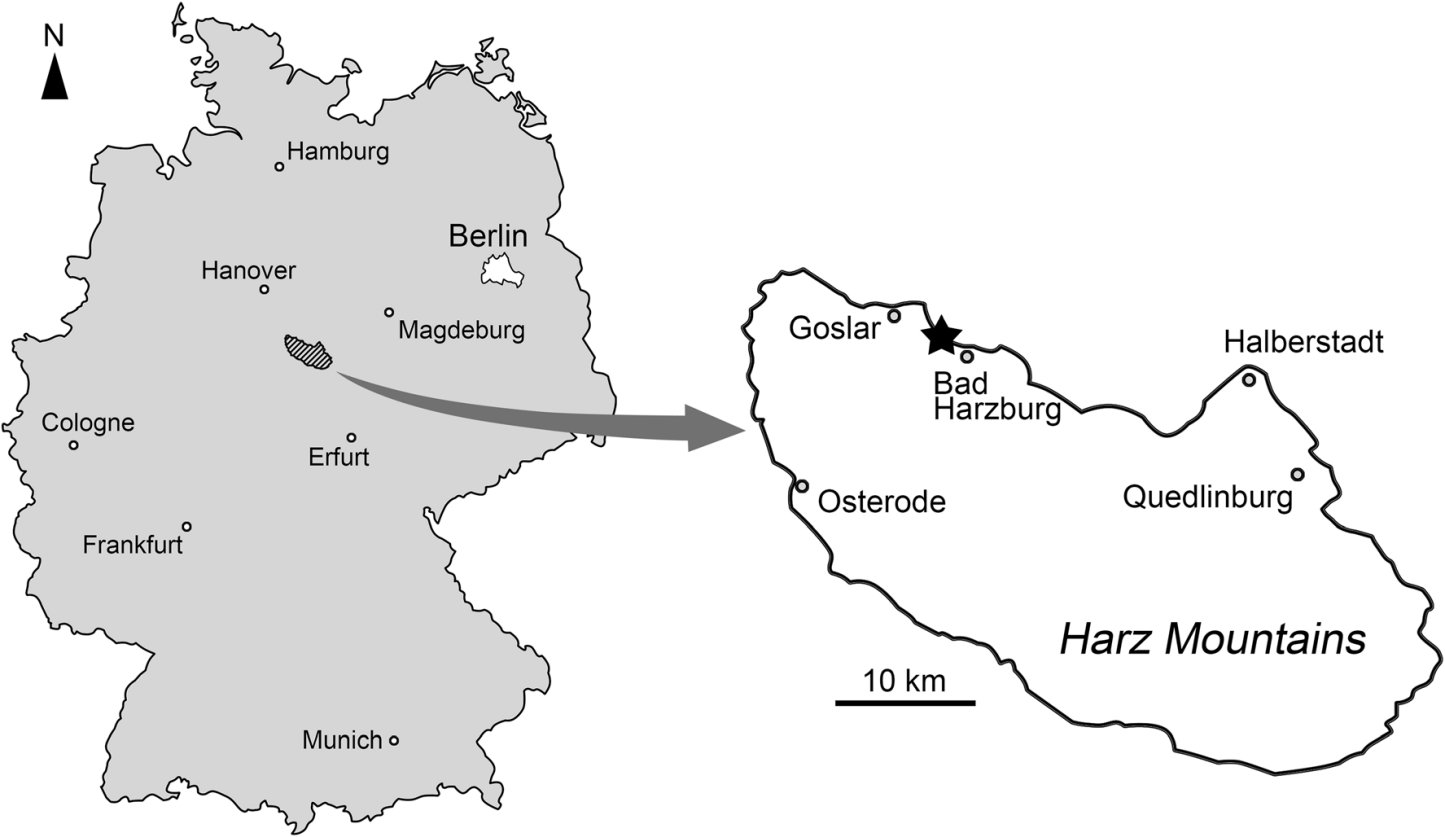
Location of the Langenberg Quarry (asterisk) at the northern rim of the Harz Mountains in northern Germany

The near shore Upper Jurassic (Kimmeridgian) marine deposits of the Süntel Formation, exposed at the Langenberg Quarry in Lower Saxony, Germany (Fig. 1), have yielded a diverse assemblage of terrestrial vertebrates, including lizards, crocodylomorphs, pterosaurs, the dwarfed sauropod dinosaur *Europasaurus holgeri*, and theropod dinosaurs (Fastnacht 2005; Sander et al. 2006; Richter et al. 2013; Carballido and Sander 2014; Lallensack et al. 2015; Marpmann et al. 2015; Gerke and Wings 2016; Schwarz et al. 2017; Carballido et al. 2020; Evers and Wings 2020). The vertebrate assemblage comprises also a number of isolated mammal teeth. Two multituberculate taxa have been described so far, *Teutonodon langenbergensis* and *Cimbriodon multituberculatus* (Martin et al. 2016, 2019b). The other mammals or mammaliaforms from this locality are the large morganucodontan *Storchodon cingulatus* (Martin et al. 2019a), a docodontan, and the new dryolestid described herein.

Dryolestidans are non-tribosphenic cladotherian mammals characterised by a specialised molar dentition and angular process of the dentary. The molars have an enhanced shearing function with subtransverse cutting edges (Schultz and Martin 2011). The upper molars are wider than the lowers, and all molars have a unilaterally hypsodont crown, which indicates a considerable rotation component of the lower jaw during the chewing cycle (Crompton et al. 1994; Schultz and Martin 2014). The lower molars have a small unicuspid talonid (without basin) and unequal roots: the distal root, supporting the talonid, is distinctly smaller than the mesial root and placed in a lingual position (e.g. Martin 1999). At least some dryolestidans differ from more advanced cladotherians by the retention of paradentary bones (coronoid and splenial) (Krebs 1969, 1971; Martin 1995). Dryolestidans have an advanced inner ear morphology with the cochlear canal coiled to at least 270° and primary and secondary bony laminae for the basilar membrane (Ruf et al. 2009; Luo et al. 2011, 2012). An almost complete postcranial skeleton is known for *Henkelotherium* from the Late Jurassic (Kimmeridgian) of Portugal, which had an arboreal lifestyle (Krebs 1991; Jäger et al. 2020). The oldest dryolestidans have been reported from the Middle Jurassic of Asia (Averianov et al. 2014), but surprisingly, this group is not known from this continent in younger deposits. The oldest European dryolestidans are from Kirtlington and are also of Middle Jurassic age (Freeman 1979). Dryolestidans were among the dominant groups of mammaliaforms in the Late Jurassic of North America and Late Jurassic to Early Cretaceous of Europe (Kielan-Jaworowska et al. 2004). The geologically youngest record of Dryolestida in the Northern Hemisphere is possibly from the Late Cretaceous (Campanian) of the USA (Lillegraven and McKenna 1986); slightly younger is the Campanian-Maastrichtian *Groebertherium* from Patagonia (Rougier et al. 2009). Here we report on a new dryolestidan genus and species from the Late Jurassic (Kimmeridgian) of Germany. Previously, in Europe, Late Jurassic dryolestidans were only known from the Iberian Peninsula (Krebs 2000; Martin 2000).

## Material and methods

The mammalian teeth from the Langenberg Quarry were recovered by screen-washing of about 10 tons of fossiliferous marly limestones from the Süntel Formation. The fossiliferous matrix was collected between 2014 and 2016 and brought to Bonn for further treatment. In the laboratory, the matrix was dissolved with 15% hydrogen peroxide and the tensid Rewoquat® (Evonik Industries AG, Essen, Germany), and subsequently screen-washed at a mesh size of 0.5 mm. After drying, microvertebrate remains were picked from the concentrate using a stereomicroscope.

The teeth were scanned with varying resolutions ranging between 3.14 and 4.03 μm using the 180 kV x-ray tube of the v|tome|x s μCT device (GE Sensing & Inspection Technologies GmbH phoenix|x-ray) housed in the Institute of Geosciences, Universität Bonn, Germany. Scan settings varied from 115 to 130 kV and 115 to 130 μA. For all scans, the same shutter speed of 400 ms per capture was used. The instrument produced isotropic voxels, and the single image size is 1024 × 1024 pixels. Avizo 8 (Thermo Fisher Scientific) was used for segmentation. The specimens are curated in Niedersächsisches Landesmuseum, Hannover, Germany (NLMH). All measurements are given in millimetres (mm).

Cladotheria were defined originally as a stem-based clade including non-symmetrodontan trechnotherians, with Dryolestoidea implicitly included in that clade (McKenna 1975). This definition was specified as a stem-based clade including all taxa more closely related to living therians than to Spalacotheriidae (Kielan-Jaworowska et al. 2004). According to a slightly different definition, Cladotheria represent a node-based taxon that includes the most recent common ancestor of dryolestidans and living therians and all its descendants (Luo et al. 2002; Martin 2018). According to these definitions, dryolestidans are not “stem cladotherians” as considered by Kielan-Jaworowska et al. (2004), but they represent early cladotherians.

**Fig. 2:**
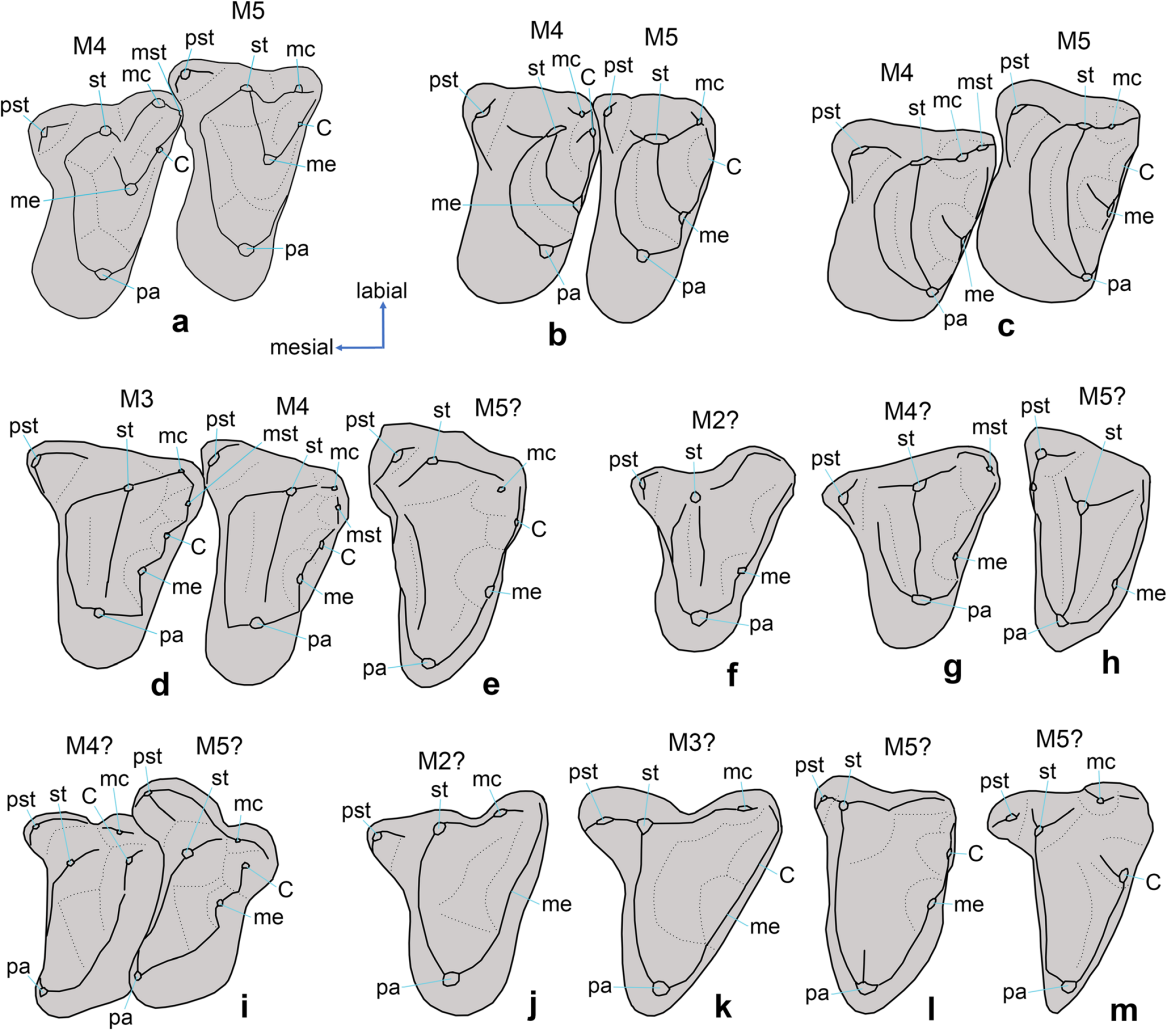
Cusp nomenclature for upper molars of selected dryolestids. (**a**) *Dryolestes priscus* (YPM 11822, mirrored); (**b**) *Dryolestes leiriensis* (Gui Mam 51/75, mirrored, modified from Martin (1999: fig. 15); (**c**) *Krebsotherium lusitanicum* (Gui Mam 73/79, modified from Martin (1999: fig. 23); (**d**) *Laolestes eminens* (AMNH 101131); (**e**) *Laolestes eminens* (USNM 2806); (**f**) *Portopinheirodon asymmetricus* (PP Mam 141/68, holotype, mirrored, modified from Martin (1999: pl. 8, fig. F); (**g**) *Laolestes andresi* (PP Mam 124/67, modified from Martin (1999: pl. 10, fig. A); (**h**) *Laolestes andresi* (PP Mam 1/67, mirrored, modified from Martin (1999: fig. 29A); (**i**) *Amblotherium gracile* (AMNH 101132, holotype of *Comotherium richi*, modified from Prothero (1981: fig. 3A); (**j**) *Crusafontia cuencana* (Uña 28, modified from Krebs (1993: fig. 1); (**k**) *Crusafontia cuencana* (MPZ CC2-1, holotype of *Crusafontia amoae*, modified after Cuenca-Bescós et al. (2011: fig. 3A); (**l**) *Crusafontia cuencana* (Galve P-2 H4, modified from Krebs (1993: fig. 2); (**m**) *Hercynodon germanicus* gen. et sp. nov. (NLMH 105668, holotype). C, metastylar cusp C; me, metacone; mc, median cusp; mst, metastyle; pa, paracone; pst, parastyle; st, stylocone. Not to scale.

There is some disagreement for the dryolestid molar cusp and crest terminology between the schemes proposed by Martin (1999: fig. 7) and Kielan-Jaworowska et al. (2004: fig. 10.2). We adopt here the following interpretations (Fig. 2): the stylocone is the largest labial cusp of the upper molars. The stylocone is connected to the paracone by the paracrista, with few exceptions, where the paracrista connects to the parastyle (e.g. Fig. 2e, g, h). In this case, the large labial cusp is called the median cusp by Kielan-Jaworowska et al. (2004: fig. 10.7C2). We call median cusp only the cusp situated between the stylocone and metastyle (Kielan-Jaworowska et al. 2004: fig. 10.2C) (Fig. 2). By this definition, the median cusp cannot exist without the presence of a stylocone, and the large labial cusp in *Laolestes andresi* is interpreted as stylocone (Fig. 2g, h ), following Martin (1999). The crest connecting paracone and stylocone (or, rarely, parastyle) is the paracrista (Martin 1999) (preparacrista in Kielan-Jaworowska et al. (2004). The crest connecting paracone and metastyle is the metacrista (Martin 1999). In Kielan-Jaworowska et al. (2004), the term metacrista is restricted to the crest between metacone and metastyle and the crest between paracone and metacone is unnamed (should be the postparacrista by homology with tribosphenic molars). In the lower molars, the crest between protoconid and metaconid is termed here protocristid instead of metacristid (Martin 1999; Kielan-Jaworowska et al. 2004), by homology with the tribosphenic lower molar (Kielan-Jaworowska et al. 2004: fig. 11.1B). The distal metacristid, a ridge extending from the metaconid apex towards the talonid (Fox 1975), is present in tribosphenic mammals and some early cladotherians, but not in dryolestidans. In dryolestidans, occasionally, there is a short vertical crest at the base of the distal side of the metaconid, termed previously distal metacristid (Averianov et al. 2014). However, as this crest is not connected with the metaconid apex, it should be named differently.

For the phylogenetic analysis, we used a short version of the data matrix presented by Averianov et al. (2014). Only Dryolestida and two outgroup taxa are included. Some characters and scorings were revised and some new characters added. In Averianov et al. (2014), *Comotherium richi* was considered a junior synonym of *Tathiodon agilis*. Here *C. richi* is considered a junior synonym of *Amblotherium gracile*. The phylogenetic position of the poorly known *T. agilis* is uncertain, and this taxon was not included in the phylogenetic analysis. *Euthlastus cordiformis* from the Late Jurassic (Kimmeridgian) of the USA was excluded from the matrix because it is more likely a more advanced, *Palaeoxonodon*-like stem therian rather than a dryolestidan. The resulting matrix comprises 17 taxa and 48 characters (Appendices 1 and 2). All characters are phylogenetically informative. *Hercynodon germanicus* gen. et sp. nov. is coded by 23 of these characters (48%). The matrix has been analysed using a heuristic parsimony tree-search algorithm that included 1000 random addition sequences (RAS), followed by tree bisection and reconnection (TBR) branch swapping and keeping 10 trees in each replication (traditional search option in TNT version 1.1 (Goloboff et al. 2008; Goloboff and Catalano 2016)). The analysis produced a single most parsimonious tree with a tree length of 68 steps, a consistency index of 0.79, and a retention index of 0.87.

3 Systematic paleontology

Mammalia Linnaeus, 1758

Cladotheria McKenna, 1975

Dryolestida Prothero, 1981

Dryolestidae Marsh, 1879

*Hercynodon* gen. nov.

urn:lsid:zoobank.org:act:9BE23710-309D-410C-BCDF-76A09FBCE7F2

Etymology. From sylva hercynia, early modern latinised name for the Harz Mountains and ὀδόν-, stem of the Greek word ὀδούς, a tooth.

Type species. *Hercynodon germanicus*, sp. nov.

Diagnosis. Referred to Dryolestidae based on combination of the following derived characters: upper molars much wider than long, large stylocone, median cusp present, metaconid more than 40% of protoconid height, and unequal roots of lower molars. Similar to *Crusafontia* but differing from other Dryolestidae by the combination of the following characters: ectoflexus on upper molars present, reduction of metacone (derived), metaconid pointed, and buccal cingulid on lower molars (derived). Differs from *Crusafontia* by a narrower crown of the upper molars (derived), a mesially less convex paracrista, and absence of a metacone (derived).

Comments. *Crusafontia cuencana* from the Lower Cretaceous (Barremian) of Spain was differentiated from all other dryolestids by the lack of the metacone (Kielan-Jaworowska et al. 2004). Actually, a crest-like metacone is present on moderately worn upper molars (Krebs 1993: figs. 1, 2). Cuenca-Bescós et al. (2011) referred two upper molars from the Galve locality (Hauterivian-Barremian) of Spain to a distinct species, *Crusafontia amoae*. However, the holotype of the latter species (Cuenca-Bescós et al. 2011: fig. 3) is nearly identical in size (the size difference is within the measurement error) and morphology with the upper molar from Uña, the type locality of *C. cuencana* (Krebs 1993: fig. 1). The only difference concerns the direction of the parastylar lobe, which is likely due to positional variation. *Crusafontia amoae* is considered here a junior subjective synonym of *Crusafontia cuencana*.

*Lakotalestes luoi*, based on the single upper molar from the Berriasian-Barremian of the USA, was interpreted originally as a dryolestid structurally closest to *Miccylotyrans minimus* [=*Amblotherium gracile*] (Cifelli et al. 2014). However, *Lakotalestes* is clearly different from *Amblotherium* and other dryolestids, including *Hercynodon* gen. nov., in having a large central cusp on the median ridge. In this respect, *Lakotalestes luoi* is similar to *Leonardus cuspidatus* from the Campanian of Argentina (Bonaparte 1990; Chornogubsky 2011) and other meridiolestidans and may be rather referred to the Meridiolestida.

Distribution. Europe, Late Jurassic (Kimmeridgian).

**Fig. 3:**
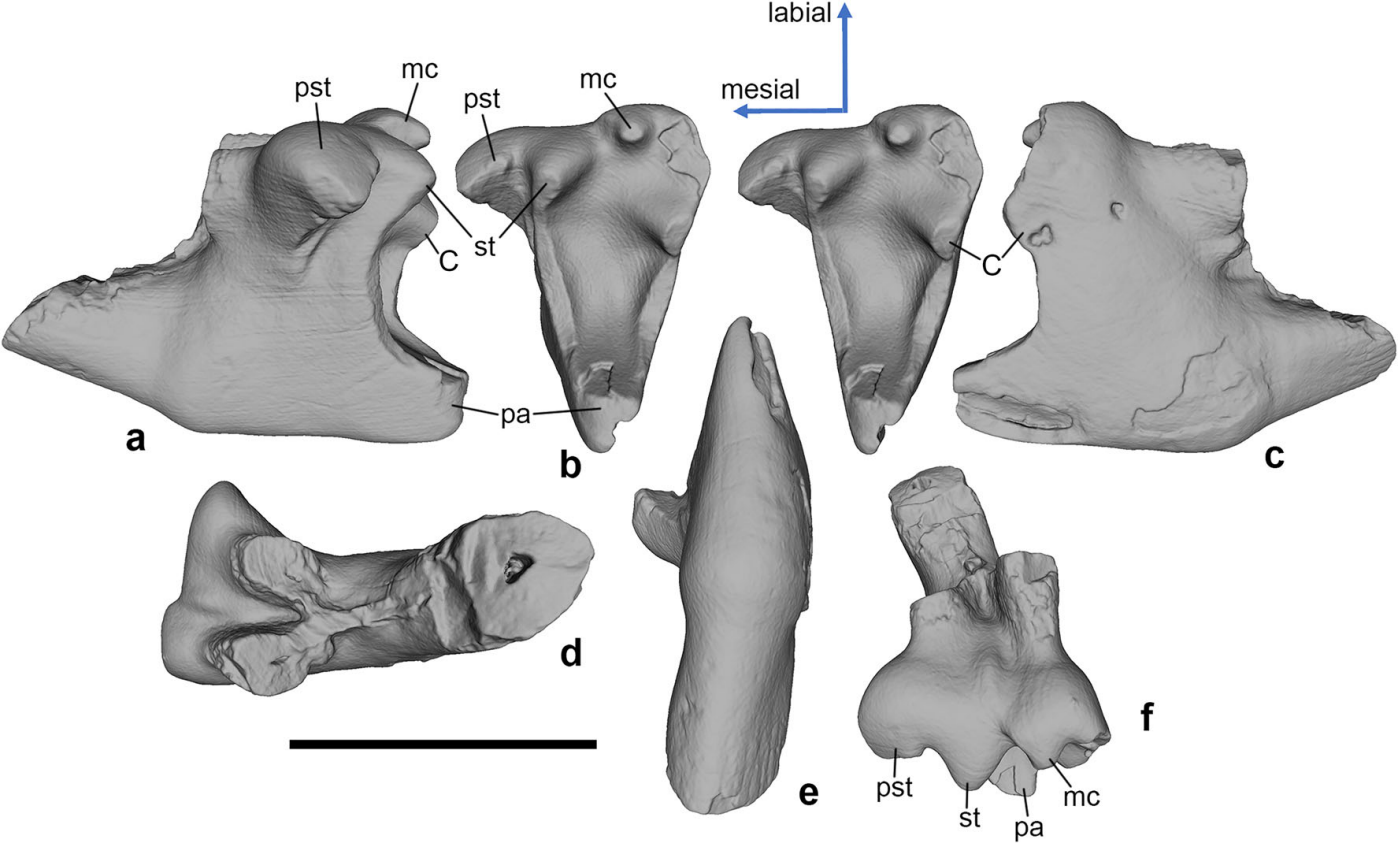
*Hercynodon germanicus* gen. et sp. nov., NLMH 105668, left upper molar (holotype). (**a**) Mesial, (**b**) occlusal (stereopair), (**c**) distal, (**d**) dorsal, (**e**) lingual, and (**f**) labial views. Langenberg Quarry, Lower Saxony, Germany; Süntel Formation, Upper Jurassic (Kimmeridgian).C, metastylar cusp C; mc, median cusp; pa, paracone; pst, parastyle; st, stylocone. Scale bar equals 1 mm.

**Fig. 4:**
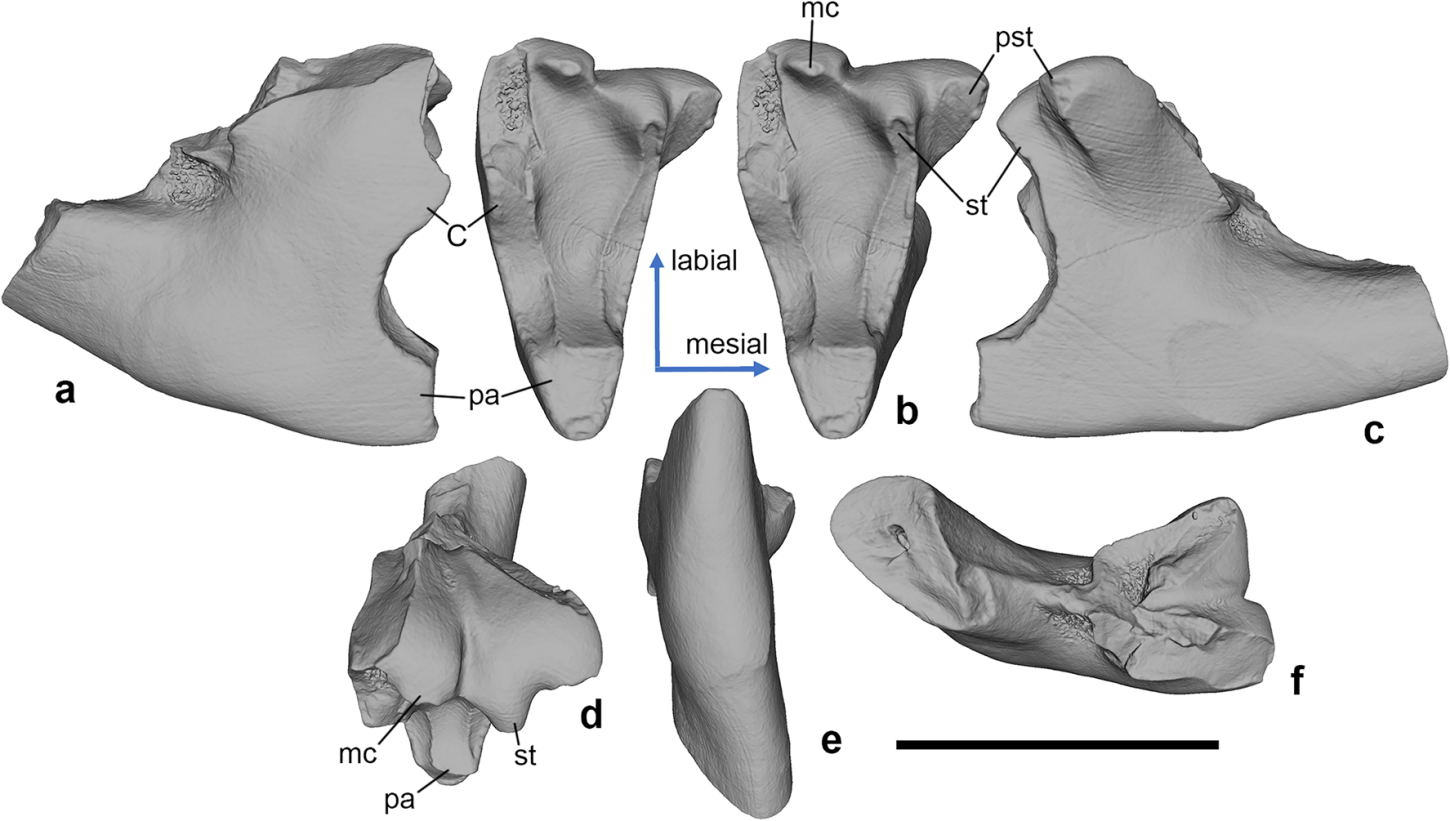
*Hercynodon germanicus* gen. et sp. nov., NLMH 105669, right upper molar. (**a**) Distal, (**b**) occlusal (stereopair), (**c**) mesial, (**d**) labial, (**e**) lingual, and (**f**) dorsal views. Langenberg Quarry, Lower Saxony, Germany; Süntel Formation, Upper Jurassic (Kimmeridgian).C, metastylar cusp C; mc, median cusp; pa, paracone; pst, parastyle; st, stylocone. Scale bar equals 1 mm.

**Fig. 5:**
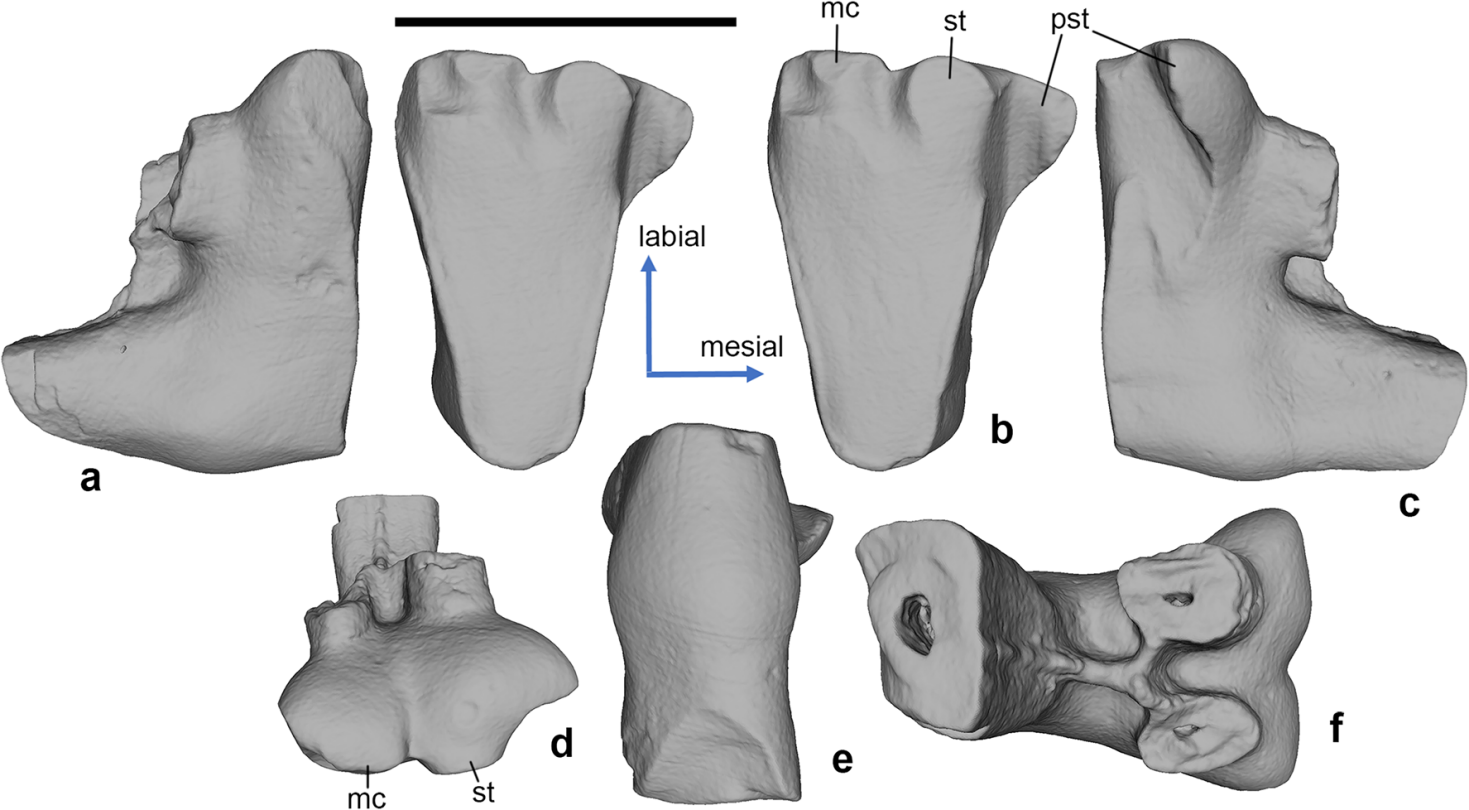
*Hercynodon germanicus* gen. et sp. nov., NLMH 105670, right upper molar. (**a**) Distal, (**b**) occlusal (stereopair), (**c**) mesial, (**d**) labial, (**e**) lingual, and (**f**) dorsal views. Langenberg Quarry, Lower Saxony, Germany; Süntel Formation, Upper Jurassic (Kimmeridgian). mc, median cusp; pst, parastyle; st, stylocone. Scale bar equals 1 mm.

**Fig. 6:**
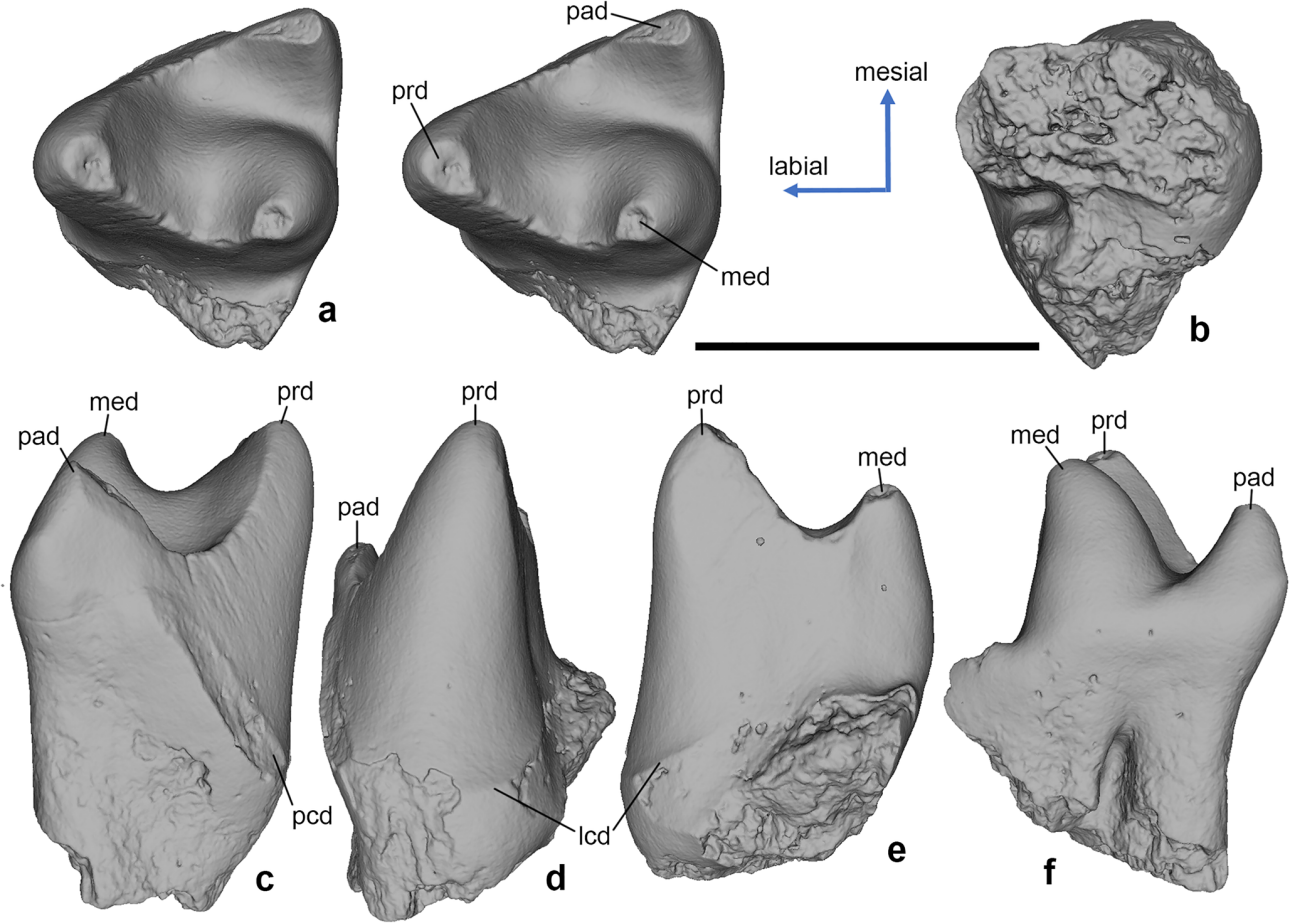
*Hercynodon germanicus* gen. et sp. nov., NLMH 105671, left lower molar. (**A**) Occlusal stereopair, (**B**) ventral, (**C**) mesial, (**D**) labial, (**E**) distal, and (**F**) lingual views. Langenberg Quarry, Lower Saxony, Germany; Süntel Formation, Upper Jurassic (Kimmeridgian). lcd, labial cingulid; med, metaconid; pad, paraconid; pcd, precingulid; prd, protoconid. Scale bar equals 1 mm.

*Hercynodon germanicus* sp. nov. (Figs. 3, 4, 5, and 6) urn:lsid:zoobank.org:pub:D79E67E1-4A25-4A4F-B124-006CF214CE88

Etymology. From Germania, Latin name for the historical region in north-central Europe where present-day Germany is located

Holotype. NLMH 105668, left upper molar.

Type locality and horizon. Langenberg Quarry near the town of Goslar, Lower Saxony, northern Germany (N 51° 54, 110′ E 10° 30, 500′). Bed 83 of Fischer (1991). The material was collected in a temporarily exposed dark lens of marl within a light grey-greenish marly limestone layer (bed 83 of Fischer 1991) within the Süntel Formation, Upper Jurassic (upper Kimmeridgian).

Referred specimens. NLMH 105669, right upper molar; NLMH 105670, left upper molar; NLMH 105671, left lower molar missing part of the talonid.

Diagnosis. As for the genus.

Description. The upper molars (Figs. 3, 4, and 5) have a unilaterally hypsodont crown, which is much higher lingually than buccally. The upper molars are narrow mesio-distally, with triangular, lingually pointed crown in occlusal view. The primary trigon angle of the little worn holotype is ~ 38° (Fig. 3B). There are three main cusps on the crown, the lingual paracone, the mesiobuccal stylocone, and the distobuccal metastylar cusp C (see “Discussion”). The paracone is much higher than the buccal cusps and somewhat damaged at the apex. The stylocone and cusp C are of similar size but cusp C is placed higher on the crown; in the holotype and NLMH 105669, it bears a linguo-distally oriented wear facet (Figs. 3b and 4b). The paracone is mesio-distally compressed, with flat buccal and convex lingual sides. Its apex is worn in all known specimens. In the holotype and NLMH 105669, mesial and distal flanks of the paracone bear faint, about 45° labio-cervically oriented striations (Figs. 3a, c and 4a, c). The paracone and cusp C are vertically oriented, whereas the stylocone is directed somewhat mesio-ventrally . Cusp C is crest-like, with convex mesial and flat distal sides. The two main shearing crests, paracrista and metacrista, are robust and connect the paracone with the stylocone and cusp C, respectively. The paracrista is longer than the metacrista because the stylocone is placed more buccally than cusp C. Both crests are worn in the holotype and NLMH 105669 (Figs. 3b and 4b). In the holotype, the wear facet of the paracrista conjoins the facet on the stylocone (Fig. 3b). The wear facet of the metacrista is separated from the facet on cusp C by a shallow notch. The deepest part of the primary trigon basin is in the middle. The buccal margin of the crown is sinusoidal, with a shallow ectoflexus. On the buccal margin of the crown, between the stylocone and cusp C, there is a median cusp, which has about half the size of the stylocone. There is a distinct short ridge on the mesiobuccal side of this cusp. This ridge connects to a larger vertical ridge on the buccal side of the crown, placed on the level between the buccal roots. There is a prominent hook-like parastylar lobe with a small but distinct parastyle. The parastyle is separated from the paracone by a distinct notch. A short precingulum extends linguo-cervically from the parastylar lobe across the half width of the crown. There are three roots, two small buccal and a large lingual, supporting the paracone. The mesial of the buccal roots is slightly smaller than the distal. The buccal roots are mesio-distally compressed, whilst the lingual root is more rounded in cross section. The bases of all three roots are connected by ridges.

In NLMH 105669, paracone, stylocone, cusp C, and the parastylar lobe are more strongly worn, with a completely worn out parastyle (Fig. 4b). The stylocone and the median cusp are more crest-like than on the holotype, and are compressed mesio-distally (stylocone) or bucco-lingually (median cusp). The vertical ridge on the buccal crown side is less pronounced than in the holotype. NLMH 105670 is heavily worn, with all the main cusps worn away (bases of stylocone and median cusp still present), and resulting flat occlusal surface (Fig. 5b). The parastylar groove is strongly worn out. Because of this extensive wear, the crown appears mesiodistally wider and less pointed lingually than in the less worn specimens.

The single known drylestid lower molar (NLMH 105671) fits in size and shape the uppers and is therefore attributed to *Hercynodon germanicus*; it lacks part of the talonid (Fig. 6). The crown is unilaterally hypsodont, much higher buccally than lingually (Fig. 6c, e). The trigonid angle is ~ 49°. The protoconid is distinctly higher than the metaconid (Fig. 6e). It has flat mesiolingual and distal sides and a convex mesiobuccal side. At the base of the mesiobuccal side of the protoconid is a slight cingulid visible which is partly missing due to corrosion (Fig. 6d, e). The distal flank of the protoconid bears faint striations that are oriented at about 45° in linguo-cervical direction (Fig. 6e). The hypoflexid groove is only slightly worn and partly corroded (Fig. 6a). The metaconid has a flat distal side, slightly convex lingual side, and strongly convex mesial side. The paraconid has about half the size of the metaconid and is crest-like, compressed mesio-distally, and tapering buccally (Fig. 6a). It has a slightly convex lingual side and flat mesial and distal sides. The mesial side bears faint, 45° linguo-cervically oriented striations (Fig. 6c). The paraconid is directed mesio-dorsally at the base but becomes more vertical towards the apex (Fig. 6f). The trigonid basin is a transverse, lingually open valley between the bases of metaconid and paraconid (Fig. 6a). The paracristid is slightly convex mesially, whereas the metacristid is convex distally. The lowest point of the paracristid is placed much lower on the crown than the lowest point of the metacristid. On the mesial crown side, there is a rather long, slightly worn, oblique precingulid, separated lingually by a shallow embayment from a poorly pronounced vertical ridge along the mesiolingual corner of the paraconid (Fig. 6c). The mesial root is bean-shaped in cross section, mesio-distally compressed. The incompletely preserved distal root has less than half the size of the mesial root (Fig. 6b, f). Both roots are connected by a pronounced interradical crest, a structure that has also been observed in the mesungulatid meridiolestid *Reigitherium* (Harper et al. 2019).

Comments. The known upper molars of *H. germanicus* sp. nov. are from a middle to more posterior tooth position. The holotype corresponds to M5 of *Achyrodon nanus* (Simpson 1928: fig. 43, pl. 10, fig. 6) or M4-5 of *Dryolestes priscus* (Simpson 1929: fig. 28). The lower molar NLMH 105671 closely matches m6 of *Crusafontia cuencana* in trigonid shape (Krebs 1993: fig. 4).

Measurements (in mm). NLMH 105668: length 0.83; width 1.12. NLMH 105669: length 0.77; width 1.15. NLMH 105670: length 0.87; width 1.22. NLMH 105671: total length 0.94 (extrapolated); trigonid length 0.74; trigonid width 0.97.

## Discussion

There is some uncertainty in interpretation of the metastylar cusp C in *Hercynodon germanicus* gen. et sp. nov. This cusp is preserved in the holotype only (Fig. 3), whereas the corresponding area is damaged or heavily worn in the two other upper molars (Figs. 4 and 5). The interpretation of the single cusp on the metacrista of *H. germanicus* gen. et sp. nov. as cusp C rather than the metacone is based on the following observations:

1) In most dryolestids, the metacone has a more lingual position, closer to the paracone (Fig. 2).

2) Cusp C of NLMH 105668 is identical in position and structure with cusp C in the posterior upper molar of *Crusafontia cuencana* (Fig. 2l), in which a poorly defined but still recognisable crest-like metacone is present, separated by a notch from cusp C. A similar notch separates cusp C from the metacrista in NLMH 105668. In more anterior upper molars of *C. cuencana*, the metacone is better pronounced and has the same position as in the posterior molar (Fig. 2j, k).

3) In the upper molar (M?5) of *Amblotherium gracile* (Fig. 2i), there is a large cusp C between the adjacent metacone and median cusp. In a more anterior upper molar of that specimen (M?4; Fig. 2i), a large cusp C and a median cusp are present, similar in size and position like in the M?5, but the metacone is lacking. This is an example of a dryolestid upper molar with a large metastylar cusp C but without a metacone. The holotype of *H. germanicus* gen. et sp. nov. is another example of this phenomenon.

**Fig. 7:**
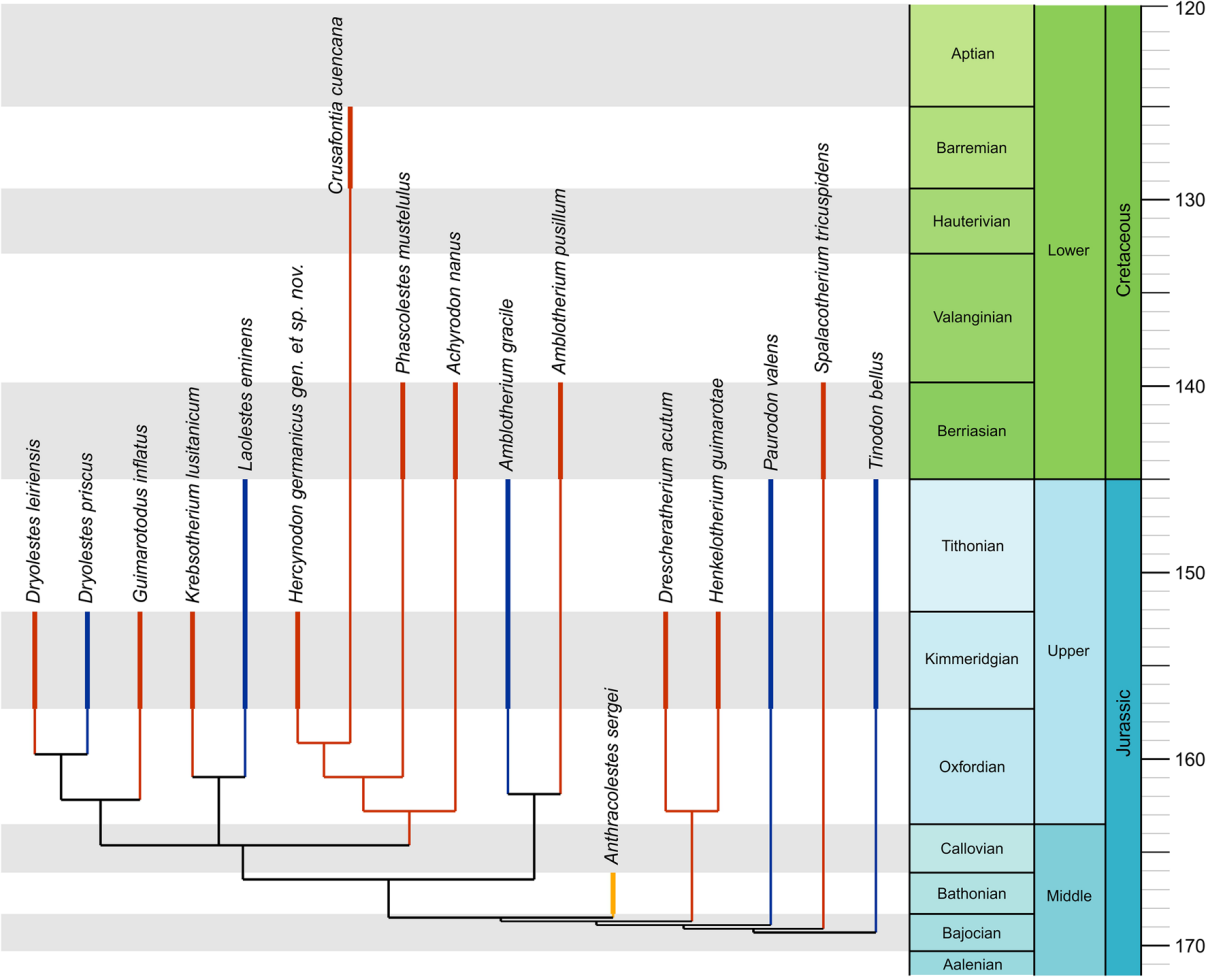
Single most parsimonious tree of Dryolestida produced by TNT traditional search analysis plotted against stratigraphical occurrence. Geographical distribution of taxa: red, Europe; blue, North America; yellow, Central Asia.

The phylogenetic analysis recovered *Hercynodon* gen. nov. as sister taxon to *Crusafontia* from the Lower Cretaceous (Hauterivian–Barremian) of Spain (Fig. 7). Both taxa are similar in reduction of the cusp pattern and enhancement of the shearing crests, paracrista and metacrista, in particular, and in reduction of the metacone. A large median cusp, much better developed than in most other dryolestids (Fig. 2), is characteristic for *Hercynodon* gen. nov. In *Crusafontia*, this cusp is variably developed, being large, similar in size with that of *Hercynodon* gen. nov. in specimen Uña 28 (Fig. 2j), but poorly differentiated in two other specimens (Fig. 2k, l). The narrow crown of the holotype and referred specimen NLMH 105669 of *H. germanicus* gen. et sp. nov. is likely a character of M5 and the more posterior molars (both specimens are interpreted as possible M5s). However, in a supposed similarly worn M5 of *Crusafontia*, the crown is somewhat wider (Fig. 2l).

*Hercynodon* gen. nov. and *Crusafontia* belong to an endemic European clade of dryolestids, including also *Achyrodon* and *Phascolestes* from the earliest Cretaceous (Berriasian) of England (Averianov et al. 2013). This clade is characterised by a reduction of the cingula on the premolars (not known for *Hercynodon*) and of the metacone on the upper molars, which is small and crest-like or absent. The two other clades of Dryolestidae are western Laurasian (Europe and North America) in distribution. The Late Jurassic *Hercynodon* gen. nov. is the oldest representative of this endemic European clade of Dryolestidae; the other taxa are of Early Cretaceous age. In spite of this, this taxon, as currently understood, is the most derived member of this clade, with completely reduced metacone. This inconsistency of geological age and morphological development may reflect an endemic evolution with increased rate of character evolution in dryolestids on temporarily isolated islands of the Late Jurassic–Early Cretaceous European archipelago (Colorado Plateau Geosystems 2012). Other insular phenomena such as insular dwarfing and possible gigantism have been described for some Late Jurassic vertebrates from the Langenberg Quarry. The small sauropod *Europasaurus holgeri* is secondarily dwarfed according to bone histological study (Sander et al. 2006). Martin et al. (2019a) noticed the large size of the morganucodontan *Storchodon cingulatus* from the Langenberg Quarry, which is the second-largest morganucodontan known from the fossil record. Large size characterises also the pinheirodontid multituberculate *Teutonodon langenbergensis* from that locality which represents the largest Jurassic multituberculate recovered so far (Martin et al. 2016, 2019b). These earlier observations, together with the discrepancy between advanced character evolution and geological age in *Hercynodon*, add to the growing body of evidence that Late Jurassic terrestrial vertebrate evolution in Europe was affected by phenomena of isolation on individual small palaeo-islands of the European archipelago. Using palaeomaps by Ziegler (1990), Sander et al. (2006) calculated that the largest palaeo-islands surrounding the Langenberg Quarry locality in the Lower Saxony Basin had areas of less than 200,000 km^2^ which would not have been able to support large-bodied sauropods. Currently, the Late Jurassic mammal assemblage from the Langenberg Quarry is still incompletely known and this hypothesis needs to be tested by new fossils.

### Supplementary Information

ESM 1 (NEX 14 kb)

## Appendix 1. List of characters.

1. Lower canine, distal cusp or short labial cingulid: absent (0), present (1).

2. Lower canine, lingual cingulid: absent (0), present (1).

3. Lower canine, number of roots: one (0), two (1).

4. Ultimate upper premolar: similar in length or shorter and narrower than M1 (0), robust, about twice longer and similar in width with M1 (1).

5. Ultimate upper premolar, cingulum: complete around the crown (0), only labial cingulum (1), no cingulum (2).

6. Lower premolars, number: three or less (0), four (1).

7. Lower premolars, relative size: gradual increase in size posteriorly (0), p2 smaller than p1 and p3 (1), p1-2 similar in size and distinctly smaller than p3-4 (2).

8. Lower premolars, anterior accessory cusp: present (0), absent (1).

9. Lower premolars, lingual cingulid: continuous (0), incomplete or absent (1).

10. Ultimate lower premolar, size relative to first molar: lower or subequal (0), taller (1).

11. Ultimate lower premolar, W/L ratio: 0.50 or less (0), between 0.51 and 0.70 (1), 0.71 or greater (2).

12. Ultimate lower premolar, mesial edge: straight (0), convex (1).

13. Ultimate lower premolar, distinct distal cingulid cusp d: absent (0), present (1).

14. Ultimate lower premolar cusp c (between main cusp a and cingulid cusp d): present (0), absent (1).

15. Upper molars, number: three or less (0), five (1), six (2), seven or eight (3).

16. Upper molar shape (calculated on widest molar in series when possible): as long as wide, or longer (L/W > 0.99) (0), wider than long (0.75 < L/W < 0.99) (1), much wider than long (L/W < 0.75) (2).

17. Upper molars, labial cingulum: present (0), absent (1).

18. Upper molars, ectoflexus: present (0), absent (1).

19. Upper molars, cusp B: present (0), absent (1).

20. Upper molars, preparacrista recurved distally towards large central stylocone: absent (0), present (1).

21. Upper molars, stylocone, size relative to metacone (or paracone, if metacone absent): smaller (0), subequal or larger (1).

22. Upper molars, median cusp: absent (0), present (1).

23. Upper molars, median crest between stylocone and paracone: absent (0), present (1).

24. Upper molars, crest between stylocone and metacone: absent (0), present (1).

25. Upper molars, metacone: large, with convex mesial side (0), small, crest-like or absent (1).

26. Upper molars, lingual cingulum: present (0), absent (1).

27. Upper molars, number of roots: two (0), three (1).

28. Lower molars, number: five or less (0), six-seven (1), eight-nine (2).

29. Lower molars, unilateral hypsodonty: absent (0), present (1).

30. Lower molars, length to width ratio (L/W): more than 1.1 (0), 1.1 or less (1).

31. Lower molars, metaconid to protoconid height: less than 40% (0), more than 40% (1).

32. Lower molars, paracristid and protocristid, lowest point: approximately at the same level (0), distinctly higher on protocristid (1).

33. Lower molars, protocristid orientation: oblique (0), transverse (1).

34. Lower molars, paraconid, direction: almost vertical, parallel to protoconid (0), mesiodorsally directed, deviated from protoconid (1).

35. Lower molars, paraconid, size relative to metaconid: subequal (0), about twice shorter (1).

36. Lower molars, metaconid: pointed (0), blunt, or chisel-like, or bifid (1).

37. Lower molars, talonid: absent or cingulid cusp d (0), heel-like (1), shelf-like (2).

38. Lower molars, lingual cingulid: present (0), absent (1).

39. Lower molars, labial cingulid: absent or incomplete (0), complete (1).

40. Lower molars, extensive wear in hypoflexid (facet 3): absent (0), present (1).

41. Lower molars, roots: equal (0), distal root much smaller and lingually placed (1).

42. Anterior end of dentary upturned dorsally so the incisors placed above the level of molars: absent (0), present (1).

43. Height of mandibular ramus between canine and last molariform: sub-uniform (1), becomes higher posteriorly (1).

44. Dentary, height of labial alveolar border relative to the lingual border: at least 80% (0), less than 80% (1).

45. Coronoid process: anteroposteriorly broad (0), narrow, separated from condylar process by deep mandibular notch (1).

46. Angle of coronoid process to toothrow: 116-135 degrees (0), 95-115 degrees (1).

47. Pterygoid crest: present, reaching dentary condyle (0), present, extending to base of angular process (1), absent (2).

48. Angular process: absent (0), present (1).

## Appendix 2. Data matrix.

*Tinodon bellus*0010?000100000?00?000000000000000000000000000100

*Spalacotherium tricuspidens*0?10000000?0002100000000000110000000001000000000

*Paurodon valens*000??001011101?10010000001100001110011010000?011

*Henkelotherium guimarotae*???011?101?10111001000000111??11110011?10?1?0011

*Drescheratherium acutum*???01?????????1100100000011?????????????????????

*Anthracolestes sergei*??1???0?????????????????????1011011021011001?0??

*Amblotherium pusillum*1?01?10101?101?2?0101100?11211111110211110111011

*Amblotherium gracile*1?1??1?1011101?200101100011211111110211110111011

*Achyrodon nanus*1?11211111?1013210101?0011121?11111121011011?111

*Phascolestes mustelulus*00012121111111321010110011121?111111210111110111

*Laolestes eminens*11110111011101321[0 1]111110011211111111210110110011

*Dryolestes priscus*???1?1?101210132111111010112111111112101101100?1

*Dryolestes leiriensis*111101110121013211111101011211111111210110110011

*Krebsotherium lusitanicum*111111110111013211111110011211111111210110110011

*Guimarotodus inflatus*111??111012101?????????????211111111210110110?11

*Crusafontia cuencana*101??121111111?210101100111211111110211111110111

*Hercynodon germanicus*???????????????210101100111?1?111110?1111???????

## Abbreviations

AMNH: American Museum of Natural History, New York, USA
MPZ: Museo de Paleontología de la Universidad de Zaragoza, Zaragoza, Spain
NLMH: Niedersächsisches Landesmuseum, Hannover, Germany
USNM: United States National Museum of Natural History, Washington, USA
YPM: Yale Peabody Museum, New Haven, USA

## References

[CR1] Averianov AO, Martin T, Lopatin AV (2013). A new phylogeny for basal Trechnotheria and Cladotheria and affinities of South American endemic Late Cretaceous mammals. Naturwisssenschaften.

[CR2] Averianov AO, Martin T, Lopatin AV (2014). The oldest dryolestid mammal from the Middle Jurassic of Siberia. J Vertebr Paleontol.

[CR3] Bonaparte JF (1990). New Late Cretaceous mammals from the Los Alamitos Formation, Northern Patagonia. Natl Geogr Res.

[CR4] Carballido JL, Sander PM (2014). Postcranial axial skeleton of *Europasaurus holgeri* (Dinosauria, Sauropoda) from the Upper Jurassic of Germany: implications for sauropod ontogeny and phylogenetic relationships of basal Macronaria. J Syst Palaeontol.

[CR5] Carballido JL, Scheil M, Knötschke N, Sander PM (2020). The appendicular skeleton of the dwarf macronarian sauropod *Europasaurus holgeri* from the Late Jurassic of Germany and a re-evaluation of its systematic affinities. J Syst Palaeontol.

[CR6] Chornogubsky L (2011). New remains of the dryolestoid mammal *Leonardus cuspidatus* from the Los Alamitos Formation (Late Cretaceous, Argentina). Paläontol Z.

[CR7] Cifelli RL, Davis BM, Sames B (2014). Earliest Cretaceous mammals from the western United States. Acta Palaeontol Pol.

[CR8] Colorado Plateau Geosystems Inc. (2012) Jurassic ca. 150 Ma. https://deeptimemaps.com/europe-series-thumbnails/. Accessed 2020

[CR9] Crompton AW, Wood CB, Stern DN, Bels L, Chardon M, Vandewalle P (1994). Differential wear of enamel: a mechanism for maintaining sharp cutting edges. Early mammals. Advances in Comparative and Environmental Physiology.

[CR10] Cuenca-Bescós G, Badiola A, Canudo JI, Gasca JM, Moreno-Azanza M (2011). New dryolestidan mammal from the Hauterivian–Barremian transition of the Iberian Peninsula. Acta Palaeontol Pol.

[CR11] Evers SW, Wings O (2020). Late Jurassic theropod dinosaur bones from the Langenberg Quarry (Lower Saxony, Germany) provide evidence for several theropod lineages in the central European archipelago. PeerJ.

[CR12] Fastnacht M (2005). The first dsungaripterid pterosaur from the Kimmeridgian of Germany and the biomechanics of pterosaur long bones. Acta Palaeontol Pol.

[CR13] Fischer R (1991). Die Oberjura-Schichtenfolge des Langenbergs bei Oker. Arbeitskreis Paläontol Hannover.

[CR14] Fox RC (1975). Molar structure and function in the Early Cretaceous mammal *Pappotherium*: evolutionary implications for Mesozoic Theria. Can J Earth Sci.

[CR15] Freeman EF (1979). A Middle Jurassic mammal bed from Oxfordshire. Palaeontology.

[CR16] Gerke O, Wings O (2016). Multivariate and cladistic analyses of isolated teeth reveal sympatry of theropod dinosaurs in the Late Jurassic of Northern Germany. PLoS One.

[CR17] Goloboff PA, Catalano SA (2016). TNT version 1.5, including a full implementation of phylogenetic morphometrics. Cladistics.

[CR18] Goloboff PA, Farris JS, Nixon KC (2008). TNT (Tree analysis using New Technology) (BETA).

[CR19] Harper T, Parras A, Rougier GW (2019). *Reigitherium* (Meridiolestida, Mesungulatoidea) an enigematic Late Cretaceous mammal from Patagonia, Argentina: Morphology, affinities, and dental evolution. J Mamm Evol.

[CR20] Jäger KRK, Luo Z-X, Martin T (2020). Postcranial skeleton of *Henkelotherium guimarotae* (Cladotheria, Mammalia) and locomotor adaptation. J Mamm Evol.

[CR21] Kielan-Jaworowska Z, Cifelli RL, Luo Z-X (2004). Mammals from the age of dinosaurs: origins, evolution, and structure.

[CR22] Krebs B (1969). Nachweis eines rudimentären Coronoids im Unterkiefer der Pantotheria (Mammalia). Paläont Z.

[CR23] Krebs B (1971) Evolution of the mandible and lower dentition in dryolestids (Pantotheria, Mammalia). In: Kermack DM, Kermack KA (eds) Early ,mammals. Zool J Linnean Soc 50, Supplement 1:89-102

[CR24] Krebs B (1991). Das Skelett von *Henkelotherium guimarotae* gen. et sp. nov. (Eupantotheria, Mammalia) aus dem Oberen Jura von Portugal. Berliner geowiss Abh A.

[CR25] Krebs B (1993). Das Gebiß von *Crusafontia* (Eupantotheria, Mammalia) - Funde aus der Unter-Kreide von Galve und Uña (Spanien). Berliner geowiss Abh E.

[CR26] Krebs B, Martin T, Krebs B (2000). The henkelotheriids from the Guimarota Mine. Guimarota: a Jurassic ecosystem.

[CR27] Lallensack JN, Sander PM, Knötschke N, Wings O (2015). Dinosaur tracks from the Langenberg Quarry (Late Jurassic, Germany) reconstructed with historical photogrammetry: evidence for large theropods soon after insular dwarfism. Palaeontol Electron.

[CR28] Lillegraven JA, McKenna MC (1986). Fossil mammals from the “Mesaverde” Formation (Late Cretaceous, Judithian) of the Bighorn and Wind River basins, Wyoming, with definitions of Late Cretaceous North American land-mammal “ages”. Am Mus Novit.

[CR29] Linnaeus C (1758) Systema naturae per regna tria naturae, secundum classes, ordines, genera, species, cum characteribus, differentiis, synonymis, locis. Vol. 1: Regnum animale. Editio decima, reformata. Laurentius Salvius, Stockholm.

[CR30] Luo Z-X, Kielan-Jaworowska Z, Cifelli RL (2002). In quest for a phylogeny of Mesozoic mammals. Acta Palaeontol Pol.

[CR31] Luo Z-X, Ruf I, Schultz JA, Martin T (2011). Fossil evidence on evolution of inner ear cochlea in Jurassic mammals. Proc R Soc B.

[CR32] Luo Z-X, Ruf I, Martin T (2012). The petrosal and inner ear of the Late Jurassic cladotherian mammal *Dryolestes leiriensis* and implications for ear evolution in therian mammals. Zool J Linnean Soc.

[CR33] Marpmann JS, Carballido JL, Sander PM, Knötschke N (2015). Cranial anatomy of the Late Jurassic dwarf sauropod *Europasaurus holgeri* (Dinosauria, Camarasauromorpha): ontogenetic changes and size dimorphism. J Syst Palaeontol.

[CR34] Marsh OC (1879). Notice of new Jurassic mammals. Am J Sci.

[CR35] Martin T, Sun A, Wang Y (1995). Dryolestidae from the Kimmeridge of the Guimarota coal mine (Portugal) and their implications for dryolestid systematics and phylogeny. Sixth Symposium on Mesozoic Terrestrial Ecosystems and Biota.

[CR36] Martin T (1999) Dryolestidae (Dryolestoidea, Mammalia) aus dem Oberen Jura von Portugal. Abh Senckenb Naturforsch Ges 550:1–119

[CR37] Martin T, Martin T, Krebs B (2000). The dryolestids and the primitive “peramurid” from the Guimarota Mine. Guimarota: a Jurassic ecosystem.

[CR38] Martin T (2018) Mesozoic mammals - early mammalian diversity and ecomorphological adaptations. In: Zachos FE, Asher RJ (eds) Mammalian evolution, diversity and systematics. Handbook of zoology. Mammalia. De Gruyter, Berlin, Boston, pp 199–299. 10.1515/9783110341553-006

[CR39] Martin T, Schultz JA, Schwermann AW, Wings O (2016). First Jurassic mammals of Germany: multituberculate teeth from Langenberg Quarry (Lower Saxony). Acta Palaeontol Pol.

[CR40] Martin T, Averianov AO, Jäger KRK, Schwermann AW, Wings O (2019). A large morganucodontan mammaliaform from the Late Jurassic of Germany. Foss Impr.

[CR41] Martin T, Averianov AO, Schultz JA, Schwermann AW, Wings O (2019). Late Jurassic multituberculate mammals from Langenberg Quarry (Lower Saxony, Germany) and palaeobiogeography of European Jurassic multituberculates. Hist Biol.

[CR42] McKenna MC, Luckett WP, Szalay FS (1975). Towards a phylogenetic classification of the Mammalia. Phylogeny of the primates.

[CR43] Prothero DR (1981). New Jurassic mammals from Como Bluff, Wyoming, and the interrelationships of non-tribosphenic Theria. Bull Am Mus Nat Hist.

[CR44] Richter A, Knötschke N, Kosma R, Sobral G, Wings O (2013) The first Mesozoic lizard from northern Germany (Paramacellodidae, Late Jurassic, Langenberg Quarry) and its taphonomy. Program and Abstracts, Society of Vertebrate Paleontology 73rd Annual Meeting, October 30 —November 2, 2013, Los Angeles, USA. Supplement to the online J Vertebr Paleontol, October 2013:198

[CR45] Rougier GW, Chornogubsky L, Casadio S, Arango NP, Giallombardo A (2009). Mammals from the Allen Formation, Late Cretaceous, Argentina. Cretac Res.

[CR46] Ruf I, Luo Z-X, Wible JR, Martin T (2009). Petrosal anatomy and inner ear structures of the Late Jurassic *Henkelotherium* (Mammalia, Cladotheria, Dryolestoidea): insight into the early evolution of the ear region in cladotherian mammals. J Anat.

[CR47] Sander PM, Mateus O, Laven T, Knötschke N (2006). Bone histology indicates insular dwarfism in a new Late Jurassic sauropod dinosaur. Nature.

[CR48] Schultz JA, Martin T (2011) Wear pattern and functional morphology of dryolestoid molars (Mammalia, Cladotheria). Paläont Z 85: 269–285

[CR49] Schultz JA, Martin T (2014). Function of pretribosphenic and tribosphenic mammalian molars inferred from 3D animation. Naturwissenschaften.

[CR50] Schwarz D, Raddatz M, Wings O (2017). *Knoetschkesuchus langenbergensis* gen. nov. sp. nov., a new atoposaurid crocodyliform from the Upper Jurassic Langenberg Quarry (Lower Saxony, northwestern Germany), and its relationships to *Theriosuchus*. PLoS One.

[CR51] Simpson GG (1928) A catalogue of the Mesozoic Mammalia in the Geological Department of the British Museum. British Museum (Natural History), London

[CR52] Simpson GG (1929) American Mesozoic Mammalia. Memoirs of the Peabody Museum of Yale University 3:1-235.

[CR53] Ziegler PA (1990) Geological atlas of Western and Central Europe, 2nd edn. Shell International Petroleum Company, Amsterdam

